# A Long-Term Survival Case of Acute Myeloid Leukemia With MYC-Positive Double Minute Chromosomes

**DOI:** 10.7759/cureus.89715

**Published:** 2025-08-10

**Authors:** Ryo Yoshimaru, Ayumi Kuzume, Hirotaka Nakamura, Yon-Mei Guo, SungGi Chi, Saki Nakamura, Michiko Iida, Kimihiko Kawamura, Kenta Akie, Yosuke Minami, Junichiro Yuda

**Affiliations:** 1 Department of Hematology and Oncology, National Cancer Center Hospital East, Kashiwa, JPN; 2 Department of Clinical Laboratory, National Cancer Center Hospital East, Kashiwa, JPN

**Keywords:** acute myeloid leukemia (aml), double minute chromosomes, long-term survival, myc, trisomy 4

## Abstract

Acute myeloid leukemia (AML) with double minute chromosomes (dmin) is a rare complication and has been reported to be refractory to chemotherapy, with a poor prognosis. A 65-year-old woman presented to a local hospital with chronic thyroiditis and sarcoidosis. She underwent a routine blood test that indicated 13% blasts in her peripheral blood. Bone marrow examination revealed 51% myeloblasts with Auer bodies, leading to the diagnosis of AML (French-American-British classification: M2; WHO classification: AML, NOS; AML with maturation). Chromosome examination (G-banding) showed the following karyotypes: 46,XX,2-11 dmin(4/20); 47,idem,+4(5/20); and 46,XX(11/20). Fluorescence in situ hybridization analysis demonstrated amplification of MYC. Genetic testing via leukemia chimeric screening was negative. The patient achieved complete remission (CR) with reduced-dose induction therapy (daunorubicin 50 mg/m² × 3 days; cytarabine 100 mg/m² × 7 days). After three courses of reduced-dose consolidation therapy (high-dose cytarabine 1,500 mg/m²), the patient remained in CR for 5 years and showed long-term survival. AML with amplification of MYC on dmin, combined with complex karyotypes, can acquire resistance to treatment due to the activity of other oncogenes in addition to MYC amplification. In cases with dmin, evaluation of other chromosomes and genetic abnormalities associated with poor prognosis is key to predicting outcome and determining the treatment plan.

## Introduction

Double minute chromosomes (dmin) are chromosome fragments without kinetochores that are generated from amplified genes in tumor cells. The term “double minute chromosomes” is derived from the fact that tumor cells in mid-mitosis exhibit a pair of microscopic chromosome fragments between chromosomes. The number of dmin per cell can range from a few to hundreds, and the amplification of genes in these chromosome fragments is believed to promote the uncontrolled growth of tumors [1,2]. dmin are frequently observed in solid tumors such as neuroblastomas (32%) and adrenal tumors (28%) but are rarely (0.3%-2.8%) noted in hematopoietic tumors [3]. Many cases of acute myeloid leukemia (AML) with dmin are associated with myelodysplasia and treatment-related diseases; moreover, they are linked to MYC and MLL amplification as well as complex karyotypes. AML cases with MYC amplification on dmin are often accompanied by mutations in TET2 (71%), TP53 (33%), and U2AF1 (25%), with a relatively uniform gene expression profile [4]. Some reports have indicated limited response to chemotherapy and poor long-term prognosis, whereas others have suggested that MYC amplification with complex chromosomal abnormalities is associated with rapid disease progression and poor prognosis. Furthermore, patients with a single chromosomal abnormality other than MYC amplification (e.g., trisomy 4 or single X chromosome deletion) exhibit an enhanced response to chemotherapy and have better prognosis [5-7]. dmin are not specified in the National Comprehensive Cancer Network (NCCN) guidelines or the European Leukemia Net (ELN) guidelines, and their evaluation has not yet been established. Although AML with MYC amplification on dmin is rare, its prognostic relevance has been proposed, which needs to be verified through the accumulation of cases. We report our experience with a patient with AML and MYC amplification on dmin, which is considered to have a poor prognosis, who achieved a long-term survival of five years.

## Case presentation

**Figure 1:**
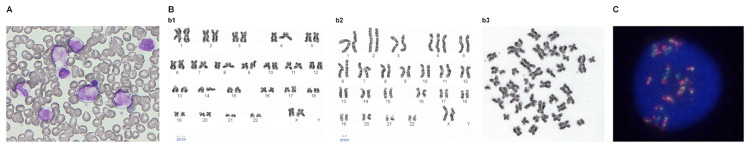
Findings of bone marrow aspiration at diagnosis (A) May–Giemsa staining showing blast cells (1000×); (B) G-banding test: (b1) 46,XX,2~11dmin(4), (b2) 47,idem,+4(5), (b3) a metaphase cell demonstrating multiple extrachromosomal double minute chromosomes (dmin); (C) fluorescence in situ hybridization analysis during interphase showing numerous MYC signals (red).

A 65-year-old woman presented to a local hospital with chronic thyroiditis and sarcoidosis. A routine blood test revealed 13% blasts in her peripheral blood. At performance status 1, she was asymptomatic, with no reported fever or bleeding tendency. Physical examination revealed no oral or cutaneous bleeding spots, enlarged lymph nodes, or hepatosplenomegaly. The findings of the blood test were as follows: white blood cell count, 3.6 × 10⁹/L (neutrophils: 56.5%, lymphocytes: 23.0%, monocytes: 4.5%, myelocytes: 0.5%, and blasts: 15.5%); hemoglobin level, 13.7 g/dL; platelet count, 269 × 10⁹/L; blood urea nitrogen level, 14 mg/dL; creatinine level, 0.67 mg/dL; aspartate aminotransferase level, 15 U/L; alanine aminotransferase level, 10 U/L; and lactate dehydrogenase level, 169 U/L, with the presence of blasts noted in the peripheral blood. However, no evidence of bone marrow suppression, liver dysfunction, or renal dysfunction was noted (Table 1). Bone marrow examination revealed hyperplastic bone marrow and 51% myeloblasts with Auer bodies (Table 2, Figure 1a). Blasts were positive for peroxidase staining and negative for esterase staining. Flow cytometry revealed that blasts were positive for CD13, CD33, CD34, HLA-DR, and CD200 and negative for cell surface markers such as B and T cells. Chromosome testing (G-banding) of the bone marrow specimen demonstrated the following karyotypes: 46,XX,2~11dmin(4/20); 47,idem,+4(5/20); and 46,XX(11/20) (Figure 1b). Fluorescence in situ hybridization (FISH) analysis showed amplification of MYC (Figure 1c). Genetic testing via leukemia chimeric screening revealed negative results for the following genes: BCR-ABL, PML-RARA, RUNX1-RUNX1T1, CBFB-MYH11, DEK-NUP214, NUP98-HOXA9, ETV6-RUNX1, TCF3-PBX1, STIL-TAL1, KMT2A-AFF1, KMT2A-AFDN, KMT2A-MLLT3, and KMT2A-MLLT1. The expression level of the Wilms tumor gene 1 was 1.5 × 10⁴ copies/μg RNA in the bone marrow. Using bone marrow specimens, gene mutation analysis (FoundationOne® Heme) revealed amplification of MYC, and no other clinically significant genetic mutations were noted.

**Table 1:** Laboratory data on admission WBC: white blood cell, Myelo: myelocyte, Seg: segmented cells, Lymph: lymphocyte, Mono: monocyte, RBC: red blood cell, Hb: hemoglobin, Hct: hematocrit, MCV: mean cell volume, Retic: reticulocyte, PLT: platelet.

Complete blood count	Value	Unit	Normal values
WBC	36×10^2^	/µL	3,000–7,800 /µL (or 3.0–7.8 ×10⁹/L)
Blast	15.5	%	0%
Myelo	0.5	%	0%
Seg	56.5	%	0-18.0%
Lymph	23	%	26.0-46.0%
Mono	4.5	%	3.0-9.0%
RBC	4.48×10^6^	/µL	3.53-4.66×10^6^/μL
Hb	13.7	g/dL	10.6-14.4g/dL
Hct	41	%	32.1-42.7%
MCV	91.5	fL	83.3-103.3 fL
Retic	16.1	‰	3-20‰
PLT	26.9×10^4^	/µL	13.8-30.9/μL

**Table 2:** Bone marrow aspirate examination Band: band cell.

Parameters	Values	Units	Normal values
M/E ratio	1.9	-	2-3
Myeloblasts	51.4	%	0.4-2.0%
Promyelocytes	0.2	%	2.0-4.0%
Myelocytes	6.2	%	8.0-15.0%
Metamyelocytes	3.6	%	7.0-22.0%
Band	3	%	9.0-15.0%
Segmented	9.4	%	6.0-12.0%
Eosinophils	0.6	%	1.0-5.0%
Basophils	0	%	0-0.4%
Monocytes	1.8	%	0-2.0%
Lymphocytes	9.8	%	10.0-18.0%
Plasma cells	1.4	%	0.4-2.0%
Erythroblasts	12.6	%	14.1-36.0%

The patient was diagnosed with AML (FAB (French-American-British) classification: M2; WHO classification: AML, NOS; AML with maturation). She received reduced-dose induction therapy (daunorubicin 50 mg/m² × 3 days; cytarabine 100 mg/m² × 7 days) and achieved complete remission (CR) with a blast count of 2.4%. G-banding revealed a normal karyotype of 46,XX(20/20), and FISH analysis indicated the disappearance of MYC amplification. The patient underwent three courses of reduced-dose consolidation therapy (high-dose cytarabine 1,500 mg/m²). Bone marrow examination revealed that CR was consistently maintained following induction therapy, and G-banding and FISH analysis confirmed no amplification of the MYC signal. The patient is currently in CR and has shown long-term survival of five years after the last course of chemotherapy.

## Discussion

In the present case, G-banding revealed karyotypes of 46,XX,2-11dmin(4/20); 47,idem,+4(5/20); and 46,XX(11/20), with dmin and trisomy 4. However, no complex karyotypes or other chromosomal abnormalities associated with poor prognosis were noted. Furthermore, FISH analysis and genetic testing demonstrated amplification of MYC, with no other genetic abnormalities associated with poor prognosis. The patient achieved CR after the initial induction therapy, remained in CR after three courses of consolidation therapy, and showed long-term survival of five years.

In a previous report of 22 cases of hematopoietic tumors with dmin (AML: 18 cases, myelodysplastic syndrome: 3, and chronic myelomonocytic leukemia: 1), following initial induction therapy, one patient achieved CR, five showed a partial response, and 16 did not respond to treatment. The median survival from diagnosis was only five months, and all patients except one, who did not undergo follow-up, died of the primary disease [5]. In another report of patients with hematologic malignancies with dmin, molecular genetic testing and FISH analysis revealed MYC (8q24) amplification [8]. MYC is an oncogene encoding a transcription factor that regulates a number of genes associated with cell cycle regulation and proliferation and plays a vital role in cell metabolism, apoptosis, differentiation, cell cycle progression, and cancer pathogenesis and progression [9,10]. An immunohistochemical analysis of MYC protein expression using bone marrow specimens of patients with untreated AML (n = 265) revealed that MYC immunopositivity of ≤6% indicated superior CR duration compared with that of >6% (23 vs. 12 months) [11]. Reports have described the relationship between dmin and MYC amplification, the role of MYC as an oncogene, and the association between high MYC protein expression and poor prognosis in AML. Conversely, some reports showed no prognostic impact of dmin. Other reports have suggested that patients with dmin and simple karyotypes (three or fewer chromosomal abnormalities) have a better prognosis than those with dmin and complex karyotypes. Moreover, cases of trisomy 4 and dmin alone are more common in women with FAB classification AML-M2 or -M4 subtype, often with MYC amplification, and they have a better prognosis than patients with dmin with other cytogenetic abnormalities [5,6,12]. Studies using cell lines and animal models have shown that MYC overexpression alone is not sufficient to induce cell transformation or tumor development; these events require the combined activity of MYC and at least one other oncogene (e.g., RAS) [13]. Thus, it is speculated that the high malignancy of leukemic cells in AML cases with complex abnormal karyotype and MYC amplification is attributed not only to MYC amplification but also to additional activation of other oncogenes and loss of tumor suppressor genes [6].

Here, we discussed the treatment strategy for patients with AML and MYC amplification on dmin. A patient with 45,X,-Y,5~33dmin(20/20) chromosomes at diagnosis who showed MYC amplification based on FISH achieved CR with daunorubicin (50 mg/m^2^ × 5 days) and cytarabine (100 mg/m^2^ × 7 days). However, the patient died of sepsis during consolidation therapy while being prepared for allogeneic hematopoietic stem cell transplantation [12]. In addition, a patient with 46,XX,3~45dmin(14/20) chromosomes and MYC signal detected by FISH achieved CR with venetoclax + azacitidine therapy and maintained CR after four courses of therapy [14].

Chromosome karyotypes and prognosis in patients with AML and MYC amplification on dmin are presented in Table 3. As observed in this case, a trend toward better prognosis has been reported in cases with only trisomy 4 and dmin and no complex karyotype [5,6,12,14]. AML cases with MYC amplification on dmin are classified as intermediate-to-high risk in the NCCN and ELN guidelines, and allogeneic HSCT after chemotherapy is considered. However, as noted in a previous report and the present case, patients with dmin and no complex karyotype and those with trisomy 4 may have a good prognosis, and long-term survival is expected with chemotherapy alone [5,6,12,14]. Considering the risk of treatment-related death from allogeneic transplantation, a careful treatment decision should be made. AML with MYC amplification on dmin acquires resistance to treatment due to complex karyotypes and the combined activity of other oncogenes in addition to MYC amplification. However, depending on the type of coexisting gene or chromosome abnormality, long-term remission has been achieved in cases with poor prognosis as well as in cases that achieved long-term remission through standard chemotherapy.

**Table 3:** Summary of patients with acute myeloid leukemia and MYC amplification on double minute chromosome F: female, M: male, FAB class: French–American–British classification, RAEB-T: refractory anemia with excess of blasts in transformation, CMML: chronic myelomonocytic leukemia, dmin: double minutes, del: deletion, add: additional material of unknown origin, dic: dicentric, der: derivative chromosome, mar: marker chromosome, der: derivative chromosome, dup: duplication.

Case	Age	Sex	FAB class	Karyotype	Trisomy 4	Complex karyotypes	Survival (month)	Reference
1	69	M	M2	45,X,-Y(10)/45,X,-Y,1~56dmin(cp10)	-	-	17	[5]
2	51	M	M2	46,XY,del(5)(q21),2~23dmin(5)/44–47,XY,del(5)(q21),del(9)(q32),add(17)(q25),4~16dmin(cp9)/46,XY(33)	-	+	12	[5]
3	67	F	M2	45,XX,del(5)(q13),-9,9~16dmin(4)/45,XX,del(5)(q13),-9,del(9)(p21),14~16dmin(14)/45,XX,del(5)(q13),-9,+18,1~8dmin(2)	-	+	6	[5]
4	66	F	M1	52–87,XXX,+3,+4,del(5)(q15)x2,+7,dic(8;9)(q24;q21)x2,del(10)(q22q24),del(11)(p15),+12,+13,+15,+16,-17,add(17)(p12), add(18)(q23),+19,add(19)(q13)del(20)(q13.2)x2,+22,8~18dmin(9)/46,XX,del(5)(1)	+	+	1	[5]
5	24	M	M1	46,XY,del(5)(q31q35),+8,add(13)(q34),der(18;21)(q10;q10),-20,add(21)(q22),+mar(7)/41–48,idem,-Y,-14,+1~3mar,2~3dmin(cp13)	-	+	3	[5]
6	64	M	RAEB-T	47,XY,+4,1~12dmin(18)/48,idem,+22(cp2)	+	-	21	[5]
7	79	F	M4	45,X,-X(8)/44–46,X,-X,1~20dmin(cp12)	-	-	19	[5]
8	71	F	M4	45–46,XX,2~8dmin(cp4)/47,XX,+4,2~11dmin(cp5)/46–47,XX,+1–3r,2~3dmin(cp8)/46,XX(3)	+	-	6	[5]
9	60	M	RAEB-T	47,XY,+6,der(17)t(17;17)(p13;q12),15~100dmin(13)/46,XY(7)	-	-	NA	[5]
10	81	F	RAEB	47,XX,+8(2)/46,XX,0–12min,0~5dmin(14)/46,XX(4)	-	-	6	[5]
11	81	M	RAEB	43–44,XY,del(5)(q13q33),-18,-20,-21,-22,+mar(cp10)/37–45,XY,idem,-12,1~4dmin(cp10)	-	+	1	[5]
12	84	M	CMML	46,XY,add(10)(p11.2)[9]/46,XY,add(10)(p11.2),1~12dmin(11)	-	-	3	[5]
13	58	M	M1	45,X,-Y,+5,-8,del(9)(q13q32),-10,-17,+mar1,+mar2,12~28dmin(20)	-	+	NA	[6]
14	54	M	M2	45,X,-Y,del(8)(q13q22),-9,del(9)(p22),？T(17;17)(p11.2;p13),+18,dmin(18)	-	+	0.5	[6]
15	59	F	M3	42–45,XX,-5,+6,-8,add(9)(p？),del(10)(q24),der(16)t(8;16)(q22;q24),del(18)(p11),der(20)t(17;20)(q21;q11),-22,+mar,dmin(35)	-	+	1.5	[6]
16	72	F	M2	45,X,-X,add(4)(p16),del(5)(q13q35),del(8)(q21.3q24.1),del(9)(q12q32),del(17)(p11.2),dmin(20)	-	+	2	[6]
17	68	F	M2	45,XX,dup(1)(q11qter),-5,-7,-11,-17,+der(17)t(17;18)(p13;q11),+r,+mar1(8)/44,XX,-5,-7,-17,+mar1,dmin(10)	-	+	2	[6]
18	75	F	M2	46,XX(5)/45,XX,-7,der(16)t(16;20)(p13;q13),der(20)del(20)(q11q13)t(16;20)(p13;q13),dmin(19)	-	+	6	[6]
19	52	F	M2	46,XX(13)/47,XX,+4,dmin(3)	+	-	9	[6]
20	72	M	M2	46,XY,(3)/46,X,-Y,+19,dmin(5)/47,X,-Y,+6,+19,dmin(7)/47,X,-Y,hsr(9)(q13),+18,+19(7)	-	+	8	[6]
21	79	F	M2	46,XX(54)/46,XX,1~22dmin(47)/47,XX,＋4,3~8dmin(5)	+	-	>13	[6]
22	70	F	M2	46,XX(7)/46,XX,dmin(4)/47,XX,+4,dmin(21)	+	-	>45	[6]
23	62	F	M2	46,XX(3)/45,X,-X,6~266dmin(53)	-	-	26	[6]
23	68	M	M2	45,X,-Y,5~33dmin(20)	-	-	4	[12]
24	81	F	M2	46,XX,3~45dmin(14/20)	-	-	NA	[14]
25	65	F	M2	46,XX,2~11dmin(4/20)/47,idem,+4(5/20)/46,XX(11/20)	+	-	60	Our case

## Conclusions

We reported our experience with a patient with AML who showed MYC amplification on dmin. She achieved CR after receiving reduced-dose induction therapy and showed long-term survival without relapse even after consolidation therapy. AML with dmin has a poor prognosis; however, there are no established evaluations listed in the NCCN or ELN guidelines. Future studies warrant the accumulation of more cases. In AML cases with dmin, evaluation of other chromosomes and genetic abnormalities associated with poor prognosis is key to predicting prognosis and determining the treatment plan. The impact of dmin and MYC on prognosis in AML is unclear, and careful monitoring after initiation of treatment is necessary.
